# Usa1 Protein Facilitates Substrate Ubiquitylation through Two Separate Domains

**DOI:** 10.1371/journal.pone.0007604

**Published:** 2009-10-29

**Authors:** Ikjin Kim, Yue Li, Paulina Muniz, Hai Rao

**Affiliations:** Institute of Biotechnology, University of Texas Health Science Center, San Antonio, Texas, United States of America; University of Birmingham, United Kingdom

## Abstract

**Background:**

Defects in protein folding are recognized as the root of many neurodegenerative disorders. In the endoplasmic reticulum (ER), secretory proteins are subjected to a stringent quality control process to eliminate misfolded proteins by the ER-associated degradation (ERAD) pathway. A novel ERAD component Usa1 was recently identified. However, the specific role of Usa1 in ERAD remains obscure.

**Methodology/Principal Findings:**

Here, we demonstrate that Usa1 is important for substrate ubiquitylation. Furthermore, we defined key cis-elements of Usa1 essential for its degradation function. Interestingly, a putative proteasome-binding motif is dispensable for the functioning of Usa1 in ERAD. We identify two separate cytosolic domains critical for Usa1 activity in ERAD, one of which is involved in binding to the Ub-protein ligase Hrd1/Hrd3. Usa1 may have another novel role in substrate ubiquitylation that is separate from the Hrd1 association.

**Conclusions/Significance:**

We conclude that Usa1 has two important roles in ERAD substrate ubiquitylation.

## Introduction

The majority of selective proteolysis in eukaryotes is handled by the proteasome [1]. Substrates of the proteasome are often covalently modified by the ubiquitin (Ub) molecule, an abundant 76-residue protein [2], [3]. Ub is activated and transferred to the substrate via several enzymes including a Ub-activating enzyme (E1), a Ub-conjugating enzyme (E2), and a Ub-protein ligase (E3). The rate-limiting step is likely the recognition and ubiquitylation of the substrate by the E3 enzyme. The ubiquitylated substrate is then degraded by proteasome. Defects in the Ub/proteasome system can lead to cancers and neurodegenerative diseases [4].

The Ub/proteasome pathway is a part of the protein quality control system responsible for the destruction of misfolded polypeptides [5], [6]. Nearly one third of cellular proteins enter the endoplasmic reticulum (ER) on their way to various cellular destinations. The folding state of secretory proteins is actively monitored in the ER. Immature proteins are retained to fold properly by ER chaperones. To prevent toxicity by the accumulation of aberrant proteins, terminally misfolded proteins are disposed of via a process termed ER-associated protein degradation (ERAD) [5], [7]. More specifically, these malfolded proteins are returned to the cytosol and recognized by a Ub-protein ligase (E3), which decorates misfolded proteins with Ub molecules that mark the substrate for proteasome-mediated proteolysis [4]. Failure of ERAD can lead to protein aggregation and cell death.

Multiple ERAD pathways are employed to eliminate aberrant proteins [6], [8]. Recent findings suggest that at least two checkpoints are employed to sort ERAD substrates into different degradation pathways based on the location of the misfolded domain (e.g. membrane, lumen, or cytosol) and the topology of the protein [9], [10], [11]. ERAD substrates with lesions exposed in the cytosol, termed ERAD-C, are selected for degradation by the Doa10 (E3) pathway. ERAD substrates with lesions in either ER membrane (ERAD-M) or ER lumen (ERAD-L) are ubiquitylated by an E3 complex composed of Hrd1 (a RING finger containing protein) and Hrd3. Interestingly, ERAD-L requires two additional proteins resided in the ER membrane, Usa1 and Der1 [10], [11]. While Der1 is proposed to be involved in the substrate retro-translocation since it has four transmembrane domains, the specific role of Usa1 in ERAD is unknown.

In the cytosol, the ATPase Cdc48 in complex with two Ub-binding proteins Ufd1 and Npl4 recognizes Ub chains and uses its chaperone activity to extract ubiquitylated proteins out of the ER [6], [8]. How the ubiquitylated ERAD substrates are transferred to the proteasome is not clear. Some, but not all ERAD substrates require Ub receptors Rad23 and/or Rpn10 [6], [8]. Since Usa1 contains a putative proteasome binding Ub-like motif (UBL) [10], [12], [13], we considered the possibility that Usa1 may have a role in bringing the proteasome close to the ER membrane and thereby shuttling substrates to the proteasome. We show herein that the UBL motif is largely dispensable for the functioning of Usa1 in ERAD-L substrate degradation. We demonstrate that Usa1 is specifically involved in the ERAD substrate ubiquitylation step. Our deletion analysis uncovers two domains essential for Usa1 function, one of which binds the Hrd1-Hrd3 E3 complex. Our data reveal that the function of Usa1 requires its association with the Hrd1-Hrd3 E3, and further suggest that Usa1 may have another undefined role in substrate ubiquitylation.

## Results

### Usa1 regulates ERAD-L substrate ubiquitylation

**Figure 1:**
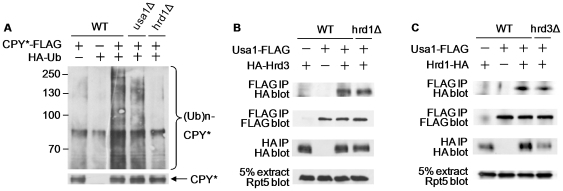
Usa1 is required for efficient substrate ubiquitylation. (A) Usa1 is involved in CPY* ubiquitylation. CPY* was immunoprecipitated from wild-type or *usa1Δ*, *hrd1Δ* cells expressing HA-tagged Ub and analyzed by immunoblotting with anti-HA antibody. The proteasome inhibitor MG132 was added to trap CPY*. Ubiquitylated CPY* are shown on the upper panel. The bottom panel shows the levels of CPY* in these cells. Molecular weight (kDa) is indicated on the left. (B) Co-immunoprecipitation analysis of interactions between Hrd3 and Usa1. The plasmid expressing Flag-tagged Usa1 was transformed into wild type or *hrd1Δ* strains expressing Hrd3 tagged with HA epitope at its genomic locus. Proteins were extracted from the indicated cells and immunoprecipitated with Flag-beads. Immunoprecipitates were separated on SDS-PAGE, and later probed with anti-HA antibody (top panel). The amounts of Usa1 and Hrd3 in cell extracts were determined and shown in lower panels. (C) Usa1 binds Hrd1 in the absence of Hrd3. Co-immunoprecipitation analysis of interactions between Usa1 and HA-tagged Hrd1 in wild-type or *hrd3Δ* cells was conducted as described in (B). Note that Hrd1 expression is reduced in *hrd3Δ* strains as previously reported [14], [15].

To determine the execution point of Usa1, we compared the ubiquitylation pattern of misfolded CPY*, a well-defined ERAD-L substrate, in wild-type and *usa1Δ* cells [9], [10], [11]. To this end, we co-transformed the plasmid expressing Flag-tagged CPY* and the plasmid bearing HA-tagged Ub to these cells. Ubiquitylated CPY* species were seen in wild-type cells expressing both CPY* and HA-Ub, but not in control cells lacking either CPY* or HA-Ub (Figure 1A). Ubiquitylated CPY* bands were significantly reduced in *usa1Δ* cells (Figure 1A), suggesting that Usa1 facilitates substrate ubiquitylation.

Usa1 was shown to bind the Hrd1-Hrd3 E3 complex [10]. Although both Hrd1 and Hrd3 are inserted into the ER membrane, their topology and functional assignments are different [6], [7]. Whereas most of Hrd3 sequences are in the ER lumen, majority of Hrd1 sequences are on the cytosolic side. In combination with two luminal proteins Yos9 and Kar2, Hrd3 recognizes misfolded substrates and targets them for ubiquitylation [11]. Hrd1, a RING finger domain protein, assembles Ub-chains onto the substrates in the cytosol. To further delineate the interaction between Usa1 and Hrd1/Hrd3, we examined these interactions in the presence or absence of one of the subunits. Usa1 binds Hrd3 (Figure 1B). In the absence of Hrd1, the interaction of Usa1-Hrd3 remained unaltered (Figure 1B). The Usa1-Hrd1 interaction was also maintained in the absence of Hrd3 albeit at slightly reduced level (Figure 1C). Note that Hrd1 level is known to be reduced in *hrd3Δ* cells [14], [15]. Combined, these results suggest that Usa1 interacts with Hrd1 and Hrd3 separately *in vivo*.

### The UBL motif is not essential for Usa1 function

**Figure 2:**
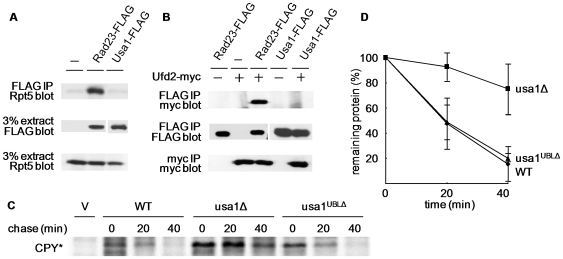
The UBL domain of Usa1 is not critical for ERAD. (A) Usa1 fails to interact with the proteasome. The plasmid expressing Flag-tagged Usa1 or Rad23 was transformed into wild type cells. To detect the association with the proteasome, the immunoprecipitates were resolved by SDS-PAGE and probed with an antibody against Rpt5, a proteasome subunit (top panel). The amounts of Rpt5, Usa1 or Rad23 in extracts are shown in lower panels. (B) Usa1 does not bind Ufd2. Proteins were extracted from cells expressing myc-tagged Ufd2 and Flag-Usa1 or Flag-Rad23. The indicated immunoprecipitation and immunoblotting were carried out as described in (A). (C) UBL domain of Usa1 is not essential for CPY* degradation. Pulse chase analysis of Flag-tagged CPY* was carried out in isogenic yeast strains BY4741 (wild-type), *usa1Δ*, YHR157 (*USA1* ^ΔUBL^). Yeast cells were pulse labeled with ^35^S for 10 min, then cold Met/Cys mix was added to start the chase. We took samples at 20 min intervals and processed them for immunoprecipitation with Flag-beads, followed by SDS-PAGE and autoradiography. (D) Quantitation of the data in C. The amount of proteins was determined by phosphor-imager analysis.

**Figure 3:**
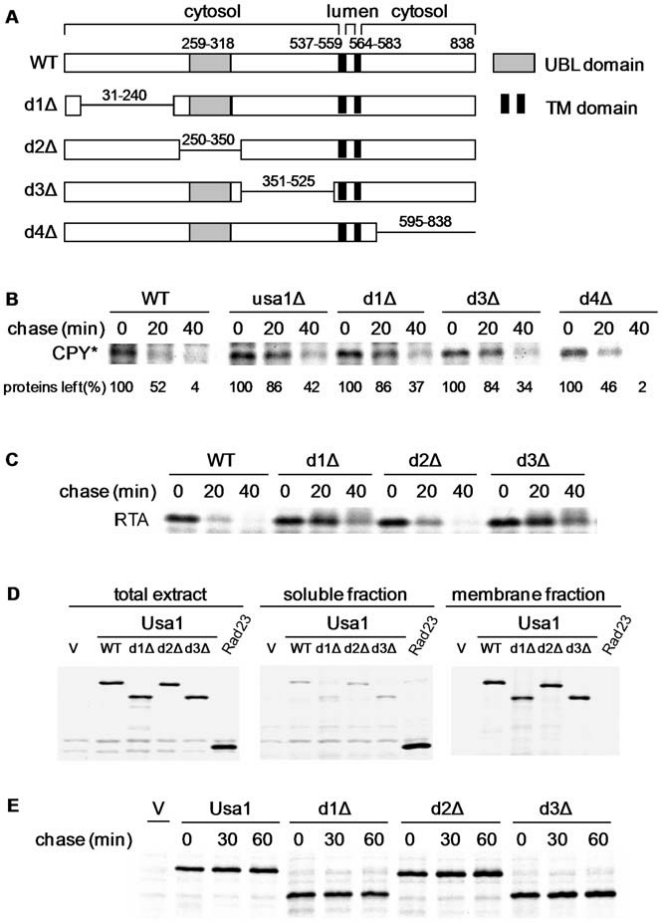
Two N-terminal fragments of Usa1 are important for ERAD. (A) Domain structure of wild-type Usa1 and various deletion mutants constructed. Two short transmembrane domains (amino acids 537–583) anchor Usa1 to the ER membrane, but most of Usa1 sequences are in the cytosol [10]. Grey box represents the UBL domain and black boxes indicate two transmembrane domains of Usa1. The deleted portions are shown as solid lines. The deleted region in d2Δ is the same as UBLΔ in Figure 2. Deletion strains and plasmids of Usa1 were constructed as described in Experimental procedures. (B) Deletions of two N-terminal segments in Usa1 impair the degradation of CPY*. Pulse chase analysis of CPY* in wild-type and various *USA1* mutant strains was performed as described in Figure 2C. The amount of CPY* left (%) is indicated under each lane. (C) Compromised degradation of glycosylated RTA in *USA1* deletion mutants. Pulse chase analysis of RTA was done as previously described [17]. (D) Usa1 deletion mutants maintain the ER membrane localization. Yeast cells expressing Flag-tagged Usa1 derivatives were labeled with ^35^S. Protein extracts were separated into total, membrane and soluble fractions and subsequently immunoprecipitated with Flag-beads. Rad23 was used as a positive control for soluble fraction. (E) Pulse chase analysis of Usa1 wild type and its derivatives. The stabilities of Flag-tagged Usa1 alleles were determined as described in Figure 2C.

**Figure 4:**
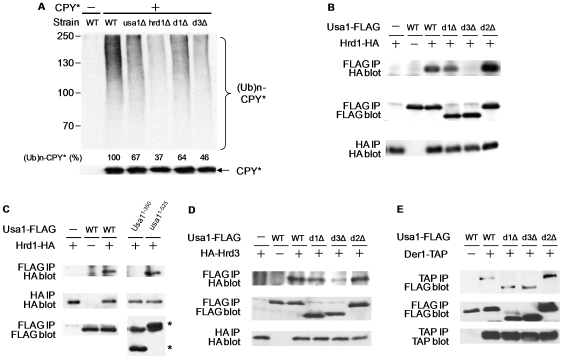
Effects of Usa1 deletions on substrate ubiquitylation and protein-protein interactions. (A) Ubiquitylation pattern of CPY* in *usa1* mutants. Flag-tagged CPY* was precipitated from wild-type or mutant cells with anti-Flag antibody and analyzed by immunoblotting with anti-Ub antibody. Molecular weight (kDa) is indicated on the left. The amount of ubiquitylated and unmodified CPY* were determined by phosphor-imager analysis. The ratio of unmodified CPY* in wild type to those in each mutant was applied to normalize the ubiquitylated CPY*. For example, the amounts of unmodified and ubiquitylated CPY in *hrd1Δ* cells are 94% and 35% of those in wild-type cells, respectively. After the normalization, ubiquitylated CPY* in *hrd1Δ* cells is 37% of that in wild-type cells. The relative amount of ubiquitylated CPY* is indicated under each lane. (B) The effects of Usa1 mutations on the interaction with Hrd1. The bindings between the indicated Usa1 derivatives and HA-tagged Hrd1 were detected by co-immunoprecipitations as described in Figure 1C. (C) The N-terminal fragment of Usa1 binds to Hrd1 without the membrane anchoring domain. Various indicated Usa1 alleles were transformed to yeast cells bearing Hrd1 tagged with HA epitope at its genomic locus. Co-immunoprecipitations were performed as described above. The positions of two Usa1 fragments are marked by asterisks. A cross-reacting band is present right below the Usa1^1–525^ fragment. (D) The interaction between Hrd3 and Usa1 derivatives. The bindings between the Usa1 mutants and HA-tagged Hrd3 were detected by co-immunoprecipitations as described in Figure 1B. (E) The effects of Usa1 mutations on Der1-binding. The plasmids bearing Usa1 derivatives were transformed to yeast strain expressing Der1 modified at its C-terminus with a Tandem Affinity Purification (TAP) tag [10]. The binding between Usa1 and Der1 was analyzed by coimmunoprecipitation (top panel). The amounts of Usa1 and Der1 in the extracts are shown in the lower panels.

The majority of Usa1 sequences is projected on the cytosolic side [10]. Besides its membrane-localization domains, the only recognizable element in Usa1 is the UBL motif, which can bind various components of the Ub/proteasome pathway including the proteasome and a Ub chain elongatoion factor Ufd2 [13], [16]. Unlike Rad23, another UBL-containing protein, Usa1 was not found in association with the proteasome or Ufd2 (Figure 2A, B). To determine the importance of this putative UBL element, we deleted the region encompassing the UBL motif (amino acids 250–350; UBLΔ also termed as d2Δ in Figures 3 and 4) at the genomic *USA1* locus. To evaluate the effect of the UBL deletion on Usa1 function, we examined the stability of two soluble ERAD-L substrates, misfolded CPY* and ricin A chain (RTA), in *usa1* mutant cells. Consistent with previous reports, CPY* is markedly stabilized in *usa1* null mutant (Figure 2C, D) [10]. Surprisingly, deletion of the UBL domain does not significantly alter the degradation of CPY* or RTA (Figure 2C, 3C), suggesting that the UBL motif is not crucial for the functioning of Usa1 in proteolysis.

### Two N-terminal domains are essential for Usa1 function in ERAD-L

To understand how Usa1 facilitates substrate ubiquitylation, we sought to identify critical sequence elements in Usa1 through mutational analysis. We deleted the C-terminal cytosolic segment (amino acids 595–838), the other two non-UBL regions of the N-terminal Usa1 (amino acids 31–240, 351–525) separately (Figure 3A) at the genomic locus of *USA1*. We found that CPY* degradation was markedly impaired in mutants lacking one of the N-terminal domains (Figure 3B), indicating that these two segments are essential for Usa1 function. In contrast, the C-terminal cytosolic tail, which accounts for nearly one third of the protein, was not required for CPY* degradation (Figure 3B) and was not further analyzed. Similarly, the turn-over of another ERAD-L substrate RTA [17] is compromised by deletions of the N-terminal, non-UBL domains (Figure 3C).

### Mutations in Usa1 do not affect its membrane localization and protein stability

Next, we examined whether the loss of function caused by these deletions was due to the change of Usa1 localization and/or stability. To facilitate the detection of Usa1 protein, Usa1 and its mutant alleles were separately fused to Flag-epitope. Yeast extracts expressing Usa1 derivatives were divided into total, soluble, and membrane fractions. Whereas the soluble Rad23 protein partitioned into supernatant portion, Usa1 mainly resides in the membrane fraction (Figure 3D). None of the N-terminal deletions affected the localization of Usa1 to the membrane (Figure 3D). Small amount of Usa1 (less than 5%) was also detected in the soluble fractions. Whether this is due to insufficient fractionation or related to its role in pre-mRNA splicing remains further investigation [18]. Then, we also carried out pulse-chase assays to measure the stabilities of Usa1 and its derivatives. Wild-type and mutant Usa1 are stable proteins (Figure 3E and data not shown).

### Defective Usa1-E3 binding affects substrate ubiquitylation

To examine the specific defects caused by these deletions, we examined the ubiquitylation pattern in *usa1* mutants. As expected, CPY* ubiquitylation was compromised in cells lacking *USA1* or *HRD1* (Figure 4A). We found reduced ubiquitylation of CPY* in two non-UBL deletion mutants (Figure 4A), suggesting that these two domains are required for substrate ubiquitylation. Interestingly, CPY* ubiquitylation is consistently more severely reduced in d3Δ mutant than in usa1 null. Although the underlying mechanism is not known, one possible explanation is that d3Δ deletion may exert a dominant negative effect since, unlike usa1 null mutant, it still has other functional domains such as the first N-terminal region and membrane anchoring domain. We suspect that these two mutations may affect the association between Usa1 and other ERAD-L ubiquitylation components. First, we determined the interaction between Usa1 derivatives and Hrd1 by co-immunoprecipitations. Interestingly, deletion of the middle portion (i.e. d3Δ) abolished the binding between Usa1 and Hrd1 (Figure 4B) and also reduced the Usa1-Hrd3 interaction (Figure 4D), suggesting that the interaction between Usa1 and the Hrd1-Hrd3 E3 complex is important for ERAD. The N-terminal fragment (amino acids 1–525) binds efficiently to Hrd1 in the absence of transmembrane domain (Figure 4C and data not shown). We also examined the binding between Usa1 and Der1. To this end, we employed Der1-TAP, which does not support ERAD but, nevertheless, associates with the other components of the Hrd1-complex [10]. None of the mutations affects the Usa1-Der1 interaction (Figure 4E). Usa1 is also known to interact with the chaperone complex Cdc48-Ufd1-Npl4 [10], which recognize and extract ubiquitylated proteins out of the ER membrane [19], [20], [21]. Since CPY* ubiquitylation is unaltered in *cdc48* mutant [19], [20], the Usa1-Cdc48 association is likely indirect and not relevant for the functioning of Usa1 in CPY* ubiquitylation. Since the d1Δ mutant retained the bindings to Hrd1, Hrd3 and Der1, the results also suggest that the extreme N-terminal domain of Usa1 plays a different, but undefined essential function in ERAD-L ubiquitylation.

## Discussion

Since the discovery of ERAD over a decade ago, the physiological significance of ERAD has been increasingly appreciated owing to its emerging, prominent role in human diseases [4], [6]. However, many important questions remain: For example, how are substrates selected for degradation? How are substrates retro-tranlocated across the ER membrane? How are substrates ubiquitylated? How are ubiquitylated substrates transferred to the proteasome? Studies in yeast have led the way in uncovering critical mechanistic attributes and the physiologic functions of ERAD. Many key players in ERAD were first identified in yeast, and nearly all of them have human counterparts [8], [11]. Assigning each ERAD factor to the specific events mentioned above presents the first step to unravel this highly coordinated choreography of ERAD.

Compromised CPY* ubiquitylation in usa1 mutants suggests the involvement of Usa1 in ERAD-L ubiquitylation. Currently, it is unknown whether Usa1 regulates substrate ubiquitylation directly or indirectly. Given the membrane barrier, ERAD-L ubiquitylation is likely much more complicated than typical ubiquitylation carried out by a normal E2–E3 pair. Besides Ubc6 E2 and Hrd1 E3, other proteins (e.g. Cue1, Hsp70 chaperones) have also been implicated in ERAD substrate ubiquitylation [22], [23]. Identification of these ubiquitylation regulators would facilitate in vitro reconstitution of ERAD ubiquitylation.

Given its ER membrane localization and mainly cytosolic topology [10], Usa1 has the potential to play multiple key roles in the ERAD pathway. Interestingly, the putative UBL motif and the C-terminal tail are not essential for ERAD-L substrate degradation (Figure 2, 3). Usa1 bridges Der1 and Hrd1 [10], which in turn could ensure the coupling of retrotranslocation and ubiquitylation. Our results also suggest that likely Usa1 has another function in ERAD in addition to its associations with Der1, Hrd1 and Hrd3 since the d1Δ mutant with largely intact bindings to Der1 and Hrd1/Hrd3 is defective for CPY* ubiquitylation (Figure 4). The specific function of the first 250 amino acids of Usa1 is unknown, but may bring other cytosolic factors required for substrate ubiquitylation close to Hrd1 [6], [7]. Our results suggest that Usa1 also works with the Hrd1–Hrd3 E3 complex to facilitate substrate ubiquitylation (Figure 4). How might Usa1 assist Hrd1 in attaching ubiquitins onto misfolded proteins? In the presence of E1 and E2, Hrd1 alone is sufficient to catalyze self-ubiquitylation *in vitro*, indicating that Hrd1 has the Ub-protein ligase activity [24]. Usa1 is likely not essential for turning on the enzymatic activity of Hrd1, but may still stimulate Hrd1 activity. It remains a possibility that Usa1 could be involved in substrate retro-translocation. Our data provides new insights regarding the mechanism governing ERAD substrate ubiquitylation and lays the foundation for uncovering the more detailed events in the ERAD pathway.

The human homologue of Usa1 is thought to be Herp since Herp partially restores the ERAD-L pathway in *usa1Δ* yeast cells [10]. Herp is induced upon ER stress and important for cell survival under stress [25], [26]. The precise function of Herp remains elusive. Like Usa1, Herp also interacts with the mammalian Hrd1–Hrd3 complex and is required for the degradation of ERAD substrates (e.g., CD3-delta, connexin 43) [26], [27], [28]. Interestingly, the only sequence similarity between Usa1 and Herp is the UBL motif [10], [25], which is essential for Herp-mediated connexin 43 degradation [26] but not for Usa1-mediated CPY* or RTA turnover (Figure 2C, 3C). Whereas Herp is an unstable protein subjected to proteasome-mediated proteolysis [26], Usa1 is quite stable (Figure 3E and data not shown). Given our findings here, it remains to be seen whether Herp and Usa1 perform a similar role in ERAD.

In summary, both two N-terminal, non-UBL domains are important for the function of Usa1 in substrate ubiquitylation. Our results lay the foundation to further uncover the detailed events involved in ERAD ubiquitylation.

## Materials and Methods

### Yeast Strains and Plasmids

Cultures were grown in rich (YPD) or synthetic media containing standard ingredients and 2% glucose (SD medium), or 2% raffinose (SR medium), or 2% galactose (SG medium), or 2% raffinose + 2% galactose (SRG medium). Wild-type strain BY4741 and isogenic mutant strains *usa1Δ*, *hrd1Δ*, *hrd3Δ*, *der1Δ* and Der1-TAP were obtained from Open Biosystems (Huntsville, AL). Yeast strains YHR161 (*USA1^Δ31–240^* in BY4741 background; d1 region deletion d1Δ), YHR157 (*USA1^Δ250–350^* ; UBL domain deletion also called d2Δ), YHR163 (*USA1^Δ351–525^*; d3 region deletion), YHR165 (*USA1^Δ595–838^*; d4 region deletion) were generated by replacing the indicated stretch with *URA3*-Myc cassette in BY4741, then *URA3* was removed by homologous recombination to leave 3 copies of myc tag in place of deleted Usa1 sequences [29]. The deletions were confirmed by PCR and DNA sequencing. Strains YTX140 (wild type), YTX297 (*HRD1*-3xHA), YRG020 (6xHA-*HRD3*), YTX485 (*HRD1*-3xHA, *hrd3Δ*), and YRG130 (6xHA-*HRD3, hrd1Δ*) are generous gifts from T. Sommer [30]. The tagged alleles of Hrd1, Hrd3, and Der1 have been shown to be functional in various protein-protein interactions and protein stability assays [30].

Flag tagged misfolded CPY* was generated in the pRS416Gal1 vector as previously described [31]. To construct the plasmid containing Flag-tagged full length Usa1 or various deletions, *USA1* gene was amplified by PCR from wild type or *USA1* deletion strains and incorporated into pRS416Gal1 vector containing Flag epitope at C-terminus. Flag-tagged RTA was previously described [17].

### Pulse-chase analysis

Pulse-chase analysis was done as described previously [17]. Yeast cells carrying plasmids that express Flag-tagged CPY*, or RTA, or Usa1 derivatives from the P*_GAL1_* promoter were grown at 30°C to an OD_600_ of ∼1 in galactose-containing medium. Cells were labeled for 10 min with 0.16 mCi of ^35^S-Express (Perkin Elmer), followed by the chase with 4 mM methionine and 2 mM cysteine. Samples were taken at the indicated time points and processed for immunoprecipitation with Flag beads (Sigma-Aldrich), followed by SDS-PAGE and autoradiography.

### Co-immunoprecipitation/immunoblotting assay

Detection of protein-protein interaction by immunoprecipitation was done as described [16], [17]. Briefly, yeast cells were grown in the SRG medium to an OD_600_ of ∼1, followed by preparation of extracts, immunoprecipitation with the beads coated with indicated antibody, SDS-8% PAGE, and immunoblotting, separately, with various antibodies.

### Detection of ubiquitylated CPY*

Analysis of CPY* ubiquitylation was carried out as described previously [32]. Briefly, CPY* was immunoprecipitated from yeast extracts treated with MG132 and the immunoprecipitates were resolved by SDS-PAGE, and immunoblotted with the antibody recognizing ubiquitin (Biomol).

### Membrane localization of Usa1 and its derivatives

Soluble and membrane fractions of yeast cells expressing either wild-type or mutant Usa1 were prepared by centrifugation [33]. Briefly, cells expressing Flag tagged Usa1 or Rad23 were labeled for 1 hour with 0.1 mCi of ^35^S-Express (Perkin Elmer), and lysed by glass beads. Cell debris was removed by centrifugation at 4,000 rpm for 5 minutes. Soluble proteins were separated from membrane fraction by centrifugation at 14,000 rpm for 10 minutes. Total extract, soluble and membrane fractions were subjected to immunoprecipitation with Flag beads, followed by SDS-8% PAGE, and autoradiography.
